# A calibration CT mini‐lung‐phantom created by 3‐D printing and subtractive manufacturing

**DOI:** 10.1002/acm2.13263

**Published:** 2021-05-05

**Authors:** H. Henry Guo, Mats Persson, Oliver Weinheimer, Jarrett Rosenberg, Terry E. Robinson, Jia Wang

**Affiliations:** ^1^ Department of Radiology Stanford Medical Center Stanford CA USA; ^2^ Department of Physics KTH Royal Institute of Technology Stockholm Sweden; ^3^ Department of Radiology University of Heidelberg Heidelberg Germany; ^4^ Department of Radiology Stanford University Stanford CA USA; ^5^ Emeritus Department of Pediatrics Stanford Medical Center Stanford CA USA; ^6^ Environmental Health and Safety Stanford University Stanford CA USA

**Keywords:** 3D printing, airway measurements, calibration, phantoms, quantitative CT

## Abstract

We describe the creation and characterization of a calibration CT mini‐lung‐phantom incorporating simulated airways and ground‐glass densities. Ten duplicate mini‐lung‐phantoms with Three‐Dimensional (3‐D) printed tubes simulating airways and gradated density polyurethane foam blocks were designed and built. Dimensional accuracy and CT numbers were measured using micro‐CT and clinical CT scanners. Micro‐CT images of airway tubes demonstrated an average dimensional variation of 0.038 mm from nominal values. The five different densities of incorporated foam blocks, simulating ground‐glass, showed mean CT numbers (±standard deviation) of −897.0 ± 1.5, −844.1 ± 1.5, −774.1 ± 2.6, −695.3 ± 1.6, and −351.0 ± 3.7 HU, respectively. Three‐Dimensional printing and subtractive manufacturing enabled rapid, cost‐effective production of ground‐truth calibration mini‐lung‐phantoms with low inter‐sample variation that can be scanned simultaneously with the patient undergoing lung quantitative CT.

## 1 INTRODUCTION

The use of quantitative CT continues to expand in lung disease, being increasingly applied to interstitial and alveolar processes such as obstruction, emphysema, fibrosis, and opacification.^1^, ^2^, ^3^, ^4^, ^5^ Measurements of airways thicknesses and parenchymal densities are vital in diffuse lung disease^6^ and recently the quantification of parenchymal opacification has been shown to predict adverse outcome in COVID‐19 pneumonia.^7^ However, uncertainty is an inherent aspect of quantification with CT imaging, being introduced by such variations as from scanner hardware, scanning protocols, reconstruction algorithms, patient body habitus, and potentially further compounded by justifiable efforts to reduce CT doses to as low as reasonably achievable (ALARA).^8^, ^9^, ^10^ Accuracy in lung density and airways measurements have been shown to be influenced by a variety of technical factors in CT acquisitions.^11^, ^12^ One strategy to reduce the aforementioned uncertainties is to scan a calibration phantom simultaneously with patients. Nelson et al. used a phantom with calcium inserts to adjust for CT number differences in coronary artery calcium CT.^13^ Henschke et al. scanned a pocket phantom embedded with Teflon sphere with patients who underwent lung cancer CT to investigate the variations in tumor volume measurement.^14^ To achieve a desired improvement in either CT attenuation quantification or geometrical assessment of pathologies, the characteristics of the calibration phantom must be tailored to the specific clinical tasks. Three‐Dimensional (3‐D) printing (also known as additive manufacturing) offers unique advantages over traditional machining techniques (also known as subtractive manufacturing). Three‐Dimensional printing facilitates rapid prototyping of complex designs, with dimensional accuracy possible to the tens of micrometers.^15^, ^16^ Three‐Dimensional printing has enabled the creation of numerous models used for education and pre‐surgical planning, and is beginning to enable CT phantom creation.^17^, ^18^, ^19^

In this work, we apply the advantages of both 3‐D printing and traditional machining to create a set of 10 duplicate calibration mini‐lung‐phantoms incorporating tubes that simulate distal airways and gradated density polyurethane foam blocks that simulate a range of parenchymal ground‐glass densities encountered in healthy and diseased lungs. The geometrical accuracy of 3‐D printed simulated airway tubes in mini‐lung‐phantoms was quantified with high‐resolution micro‐CT, and the CT number of machined ground‐glass density blocks and geometrical accuracy of airway tubes were evaluated on clinical CT scanners from two CT manufacturers.

## 2 METHODS

### 2.1 Mini‐lung‐phantom creation

**Fig. 1:**
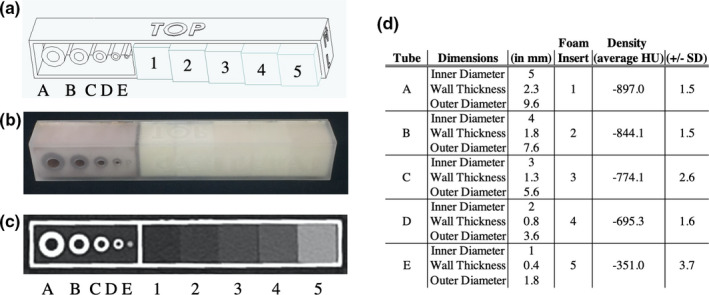
The calibration CT mini‐lung‐phantom with simulated airway tubes and ground‐glass densities. (a) Computer‐aided design schematic of the mini‐lung‐phantom, showing five tubes with designed nominal 5, 4, 3, 2, 1mm inner diameters, labeled as tube A, B, C, D, E, respectively, and five polyurethane foam inserts simulating increasing ground‐glass densities in the phantom, labeled as inserts 1, 2, 3, 4, 5, with nominal 0.096, 0.160, 0.240, 0.320, and 0.641 g/cm^3^ densities, respectively. (b) Photograph of an external calibration mini‐lung‐phantom. (c) Axial clinical CT Image of the phantom, demonstrating tubes A‐E and foam block inserts 1–5. (d) Specifications of mini‐lung‐phantoms with nominal dimensions: inner diameter (ID), wall thickness (WT), and outer diameter (OD) of tubes A‐E, and CT HU densities of foam block inserts 1–5 as listed.

No IRB review was required for this phantom study. The dimensions of the tubes simulating large to small airways (nominal inner diameters of 5, 4, 3, 2, 1 mm; with corresponding nominal outer diameters of 9.6, 7.6, 5.6, 3.6, 1.8 mm, respectively) were based on previous airways phantoms designed for dimensional measurement accuracy quality control in chest CT, with tube dimensions approximating distal bronchi and bronchioles.^20^, ^21^, ^22^ Aside from tube dimensions, the mini‐lung‐phantom is designed to be much smaller (1.75 × 1.75 × 12 cm) than conventional airways phantoms given the current intent of the mini‐lung‐phantom being placed on patients and scanned simultaneously. The mini‐lung‐phantom design was drafted by computer‐aided design (SolidWorks, Dassault Systèmes, Vélizy‐Villacoublay Cedex, France) [Fig. 1(a)]. The engineering file was translated to standard tessellation language (STL) for 3‐D printing using a Viper stereolithography machine (3D Systems, Rock Hill, SC, USA) on high‐resolution mode using Accura ClearVue resin (Autotiv Manufacturing, Salem, NH). Three‐Dimensional printing produced the shell, simulated airways tubes, and lid assembly of the mini‐lung‐phantom. As the current limitations of 3‐D printing technology prevent consistent and accurate reproduction of clinically relevant ground‐glass densities, polyurethane foam was used; and five different rigid polyurethane foam blocks from the same respective manufacturing batches with nominal densities of 0.096, 0.160, 0.240, 0.320, and 0.641 g/cm^3^ (General Plastics, Tacoma, WA, USA) were machined into 1.5 cm cubes (WeCutFoam, Sunnyvale, CA) and inserted in the described order within the shell of the 3‐D printed mini‐lung‐phantoms (numbered as foam blocks 1, 2, 3, 4, 5), respectively [Fig. 1(a)]. Polyurethane foams from the same manufacturer have been validated for use in CT scanning previously.^23^, ^24^ Phenolic micro‐balloons (MAS Epoxies, South St. Paul, MN, USA) at −905 HU density was poured into the 3‐D printed cavity adjacent to the tube containing compartment of the mini‐lung‐phantom to simulate surrounding lung density. Finally, the lid was sealed to the body of the assembly using ethyl‐2‐cyanoacrylate adhesive [Fig. 1(b)]. Ten duplicate calibration mini‐lung‐phantoms were created.

### 2.2 Micro‐CT imaging protocol

Micro‐CT scanning of all 10 mini‐lung‐phantoms was performed using an eXplore CT120 scanner (TriFoil Imaging, Chatsworth, CA, USA). Cone‐beam CT scans were performed at 2 × 2 binning, 70 kV, 40 mA, with 720 views acquired in a 360‐degree scan. Axial images were reconstructed with an isotopic 50 µm voxel size.

### 2.3 Clinical CT imaging protocols

Clinical CT scanning of the 10 duplicates of mini‐lung‐phantoms was performed inside a tissue‐density elliptical ring phantom with a lateral width of 33 cm (CT ACR 464 Phantom Body Ring, Gammex, Middleton, WI, USA). All mini‐lung‐phantoms were scanned on two clinical CT scanners (Scanner A: SOMATOM FORCE, Siemens, and scanner B: Discovery CT750 HD, GE) using our institution’s clinical quantitative chest CT protocols. Scans on scanner A were performed with the following parameters: 120 kV or 100 kV with tin filter (100Sn), 0.25 s rotation time, pitch of 1, 192 × 0.6 mm collimation. Scans were performed with three dose levels: CTDI_vol_ of 0.1 (100Sn), 1.99 (120 kV), and 6.67 (120 kV) mGy. Bf32 kernel was used for image reconstruction at 1mm thickness. Scanner B parameters were: 120 kV, 0.5 s rotation time, pitch 1.375, 64 × 0.625 mm collimation. Scans were performed at three dose values: CTDI_vol_ of 0.29, 2.04, and 6.71 mGy. “Standard” kernel was used for image reconstruction at 1.25 mm thickness. The reconstruction field‐of‐view (FOV) was 30 cm for images acquired on both scanners, using a standard 512 × 512 reconstruction matrix.

### 2.4 Dimensional analyses of simulated airway tubes

**Fig. 2:**
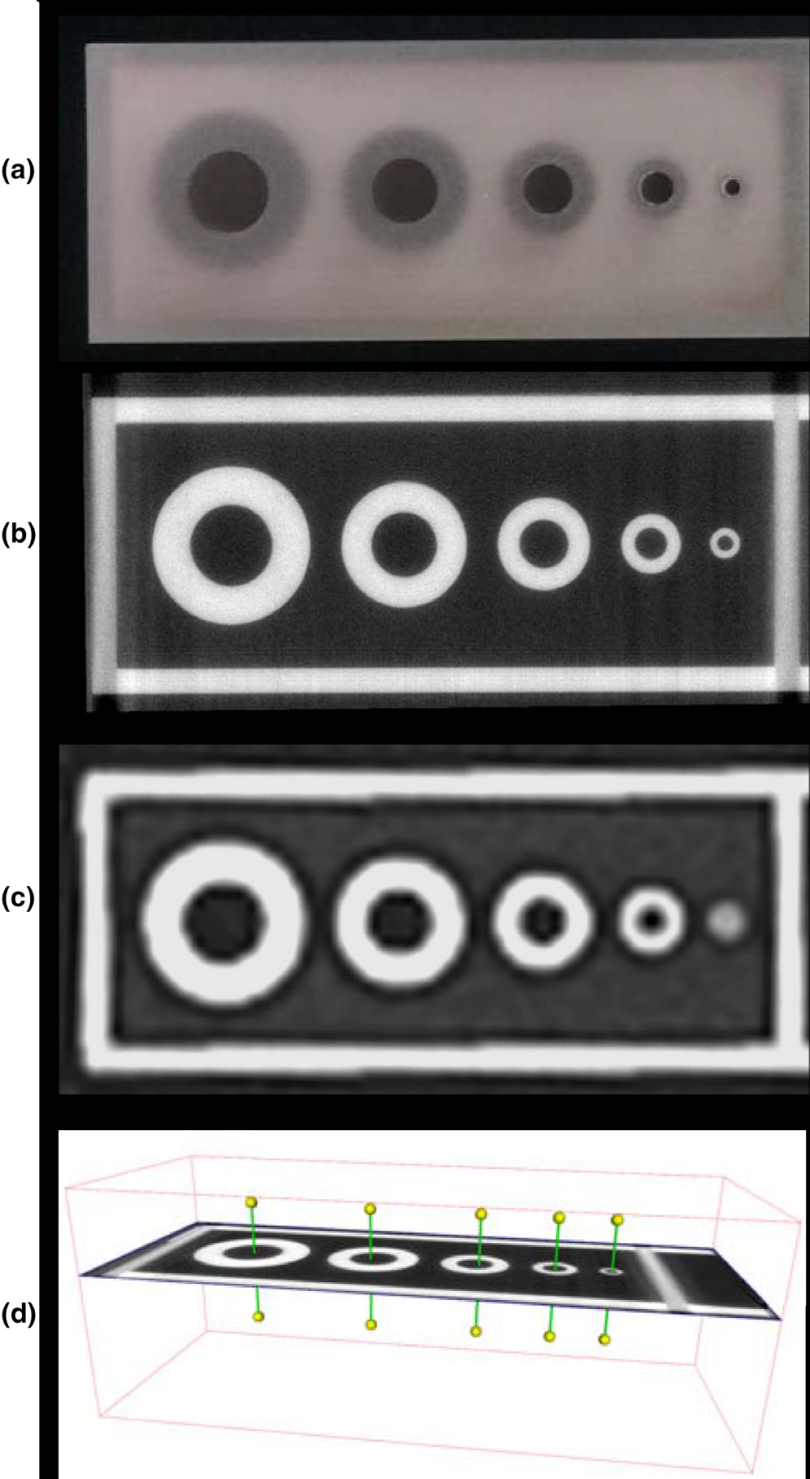
Micro‐CT and clinical CT images of the mini‐lung‐phantom and dimensional measurements of its simulated airways. (a) Close‐up photograph of the five tubes A, B, C, D, and E in a mini‐lung‐phantom. (b) Axial micro‐CT image of the five tubes, with designed nominal inner diameters of 5, 4, 3, 2, 1 mm. (c) Axial clinical CT image of the five tubes. Note that the smallest tube E with 1 mm ID is poorly resolved by the clinical CT scanner. (d) Schematic of YACTA analysis of tube dimensions from micro‐CT, with pins denoting centerlines.

Images from micro‐CT and clinical CT scanning of the tubes simulating airways were analyzed in an automated fashion using YACTA software (version 2.8.5.36).^25^ For clinical CT analysis, only images acquired from scanner A at the highest dose are investigated for airway tube dimension measurements. Segmentation of the lumen was performed by a 3D region‐growing algorithm, followed by an iterative topology‐preserving 3D thinning algorithm to produce a single skeleton line (centerline) and direction vector for each tube. Orthogonal planes for every skeleton point were calculated and tubes’ lumen and wall thicknesses were determined using the parameter‐free integral‐based method [Fig. 2(d)].^25^, ^26^, ^27^ Measured dimensions of the inner diameters (ID), wall thicknesses (WT), and outer diameters (OD) from micro‐CT and clinical CT images were compared with corresponding nominal values. To determine the inter‐sample variance from the manufacturing process, 95% confidence/99% coverage tolerance intervals of each measurement type (ID/WT/OD) were calculated for deviations across all 10 mini‐lung‐phantoms from micro‐CT images. Nonparametric (Hahn‐Meeker method) intervals were calculated using R version 3.5.3 (r‐project.org) and version 1.3.0 of the “tolerance” package.

### 2.5 Ground‐glass foam blocks density analyses

Average CT numbers were measured in a square region of interest (6.4 × 6.4 mm) in each of the foam block inserts on the clinical CT images using an in‐house developed program (MATLAB, The MathWorks, Inc., Natick, MA, USA). The measurements were then averaged over a longitudinal thickness of 7.6 mm for each block. Finally, the mean and standard deviation of block CT numbers over 10 mini‐lung‐phantoms were calculated.

## 3 RESULTS

**Fig. 3:**
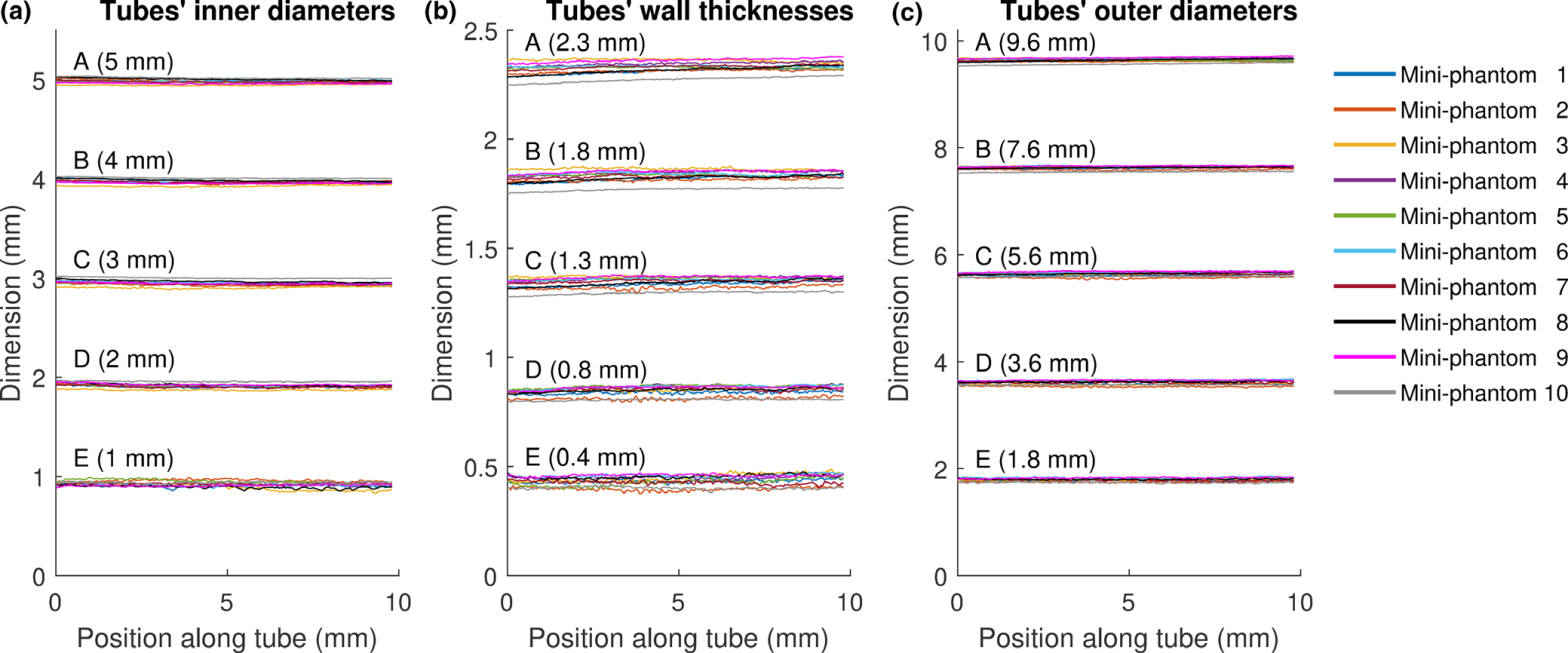
Micro‐CT measurements of (a) inner diameters, (b) wall thicknesses, and (c) outer diameters of every tube (A‐E) plotted along tubes’ long axis. The design specified nominal dimensions of ID, WT, and OD of each tube are provided in parentheses.

**Fig. 4:**
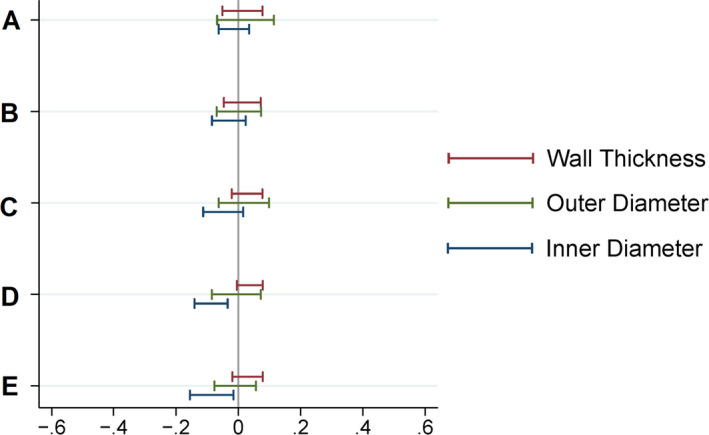
95% confidence / 99% coverage tolerance intervals for deviation from inner diameter, wall thickness, and outer diameter nominal values based on micro‐CT measurements of five tubes (A‐E) of all 10 mini‐lung‐phantoms. Such intervals are calculated to contain 95% confidence 99% of all future observations. Nonparametric (Hahn‐Meeker method) intervals were calculated. The tolerance intervals for all parameters are well within ±0.2 mm.

3D‐printed and machined components were assembled to produce 10 identical mini‐lung‐phantoms. Figure 1 shows the design schematic, photograph, clinical CT appearance, and unit dimensional and density specifications of the calibration mini‐lung‐phantom. The close‐up photo, micro‐CT, and clinical CT images of the airways mimicking tubes are shown in Fig. 2. Table 1 reports the designed (nominal) and measured dimensions of the ID, WT, and OD of the five tubes in the 10 mini‐lung‐phantoms from micro‐CT and clinical CT images from scanner A at highest dose. The conformity to nominal dimensions along the entire long axis of each tube from micro‐CT images is shown in Fig. 3. The inner diameters measured on the micro‐CT scans showed an average absolute variation of 0.016, 0.031, 0.052, 0.086, and 0.080 mm from nominal values. Overall, the average absolute variation of ID, WT, and OD from nominal values was 0.038mm. The 95% confidence / 99% coverage tolerance intervals from micro‐CT images are shown in Fig. 4. The tolerance intervals for all parameters were well within 0.2mm, which is smaller than the spatial resolution of most clinical CT scanners. Dimensional error measurement from clinical CT images of tubes demonstrated greater deviation from nominal values, with an average variation of 0.3mm (*P* = 0.0049, two‐sided Wilcoxon signed‐rank test for 12 pairs of measured dimensions from micro‐CT and clinical CT: ID, WT, and OD for tubes A‐D). Note that the smallest tube E at 1 mm inner diameter was not adequately visualized on clinical CT images (Fig. 2), consistent with expected clinical CT resolution limits, and hence not measured.

**Table 1:** Measurements of inner diameter, wall thickness, and outer diameter for the five simulated airways tubes, from A (largest) to E (smallest) from micro‐CT and clinical CT scanning (scanner A). Average variation denotes measured difference from nominal values. Tube E was not well visualized with clinical CT and hence not measured.

Tube	Measurement	Designed Nominal value (mm)	Micro‐CT average ± std. dev. (mm)	Micro‐CT average variation from nominal (mm)	Clinical CT average ± std. dev. (mm)	Clinical CT average variation from nominal (mm)
A	Inner Diameter	5	4.98 ± 0.021	−0.016	4.60 ± 0.013	−0.40
	Wall Thickness	2.3	2.33 ± 0.027	0.028	2.50 ± 0.021	0.20
	Outer Diameter	9.6	9.64 ± 0.034	0.039	9.60 ± 0.035	0.004
B	Inner Diameter	4	3.97 ± 0.021	−0.031	3.45 ± 0.032	−0.55
	Wall Thickness	1.8	1.83 ± 0.026	0.028	2.07 ± 0.042	0.27
	Outer Diameter	7.6	7.63 ± 0.035	0.026	7.60 ± 0.066	−0.002
C	Inner Diameter	3	2.95 ± 0.025	−0.052	2.37 ± 0.042	−0.63
	Wall Thickness	1.3	1.35 ± 0.024	0.046	1.59 ± 0.018	0.29
	Outer Diameter	5.6	5.64 ± 0.038	0.039	5.54 ± 0.049	−0.058
D	Inner Diameter	2	1.91 ± 0.022	−0.086	1.31 ± 0.033	−0.69
	Wall Thickness	0.8	0.85 ± 0.022	0.047	1.01 ± 0.028	0.21
	Outer Diameter	3.6	3.61 ± 0.041	0.008	3.32 ± 0.042	−0.28
E	Inner Diameter	1	0.92 ± 0.023	−0.080	NA	NA
	Wall Thickness	0.4	0.44 ± 0.022	0.035	NA	NA
	Outer Diameter	1.8	1.79 ± 0.029	−0.010	NA	NA

The CT numbers of each of the five different foam inserts measured from two clinical CT scanners are listed in Table 2. The composite mean CT numbers (HU) and standard deviation from two scanners were: −897.0 ± 1.5, −844.1 ± 1.5, −774.1 ± 2.6, −695.3 ± 1.6, −351.0 ± 3.7 HU for inserts 1–5, respectively (Fig. 1). For comparable dose levels, the average CT numbers differed by at most 3.0 HU between the two scanners (Table 2). The standard deviation of block CT numbers among the 10 duplicated phantoms ranges from 1.0 to 4.9 HU.

**Table 2:** CT numbers of five foam blocks of 10 mini‐lung‐phantoms from two clinical CT scanners at three CT dose levels. The mean and standard deviation of CT numbers for each block are averaged values over 10 duplicated mini phantoms.

	Foam block 1		Foam block 2		Foam block 3		Foam block 4		Foam block 5	
Scanner and dose (CTDI_vol_)	Mean (HU)	Std Dev (HU)	Mean (HU)	Std Dev (HU)	Mean (HU)	Std Dev (HU)	Mean (HU)	Std Dev (HU)	Mean (HU)	Std Dev (HU)
Scanner A routine dose (6.67 mGy)	−896.5	1.7	−843.3	1.5	−774.2	2.8	−695.5	1.6	−350.8	3.7
Scanner A low dose (1.99 mGy)	−896.0	2.2	−843.0	1.6	−774.7	3.0	−696.1	2.5	−351.5	4.9
Scanner A ultra−low dose (0.1 mGy)	−899.0	2.5	−844.1	4.0	−774.5	4.5	−694.5	3.3	−347.0	3.3
Scanner B routine dose (6.71 mGy)	−897.4	1.2	−844.9	1.0	−773.9	2.6	−695.1	1.7	−351.3	3.9
Scanner B low dose (2.04 mGy)	−897.4	1.5	−845.0	1.2	−774.3	2.5	−694.7	2.1	−350.9	4.1
Scanner B ultra‐low dose (0.29 mGy)	−897.1	3.3	−845.1	1.6	−774.7	3.2	−694.6	3.4	−350.0	3.9

## 4 DISCUSSION

**Fig. 5:**
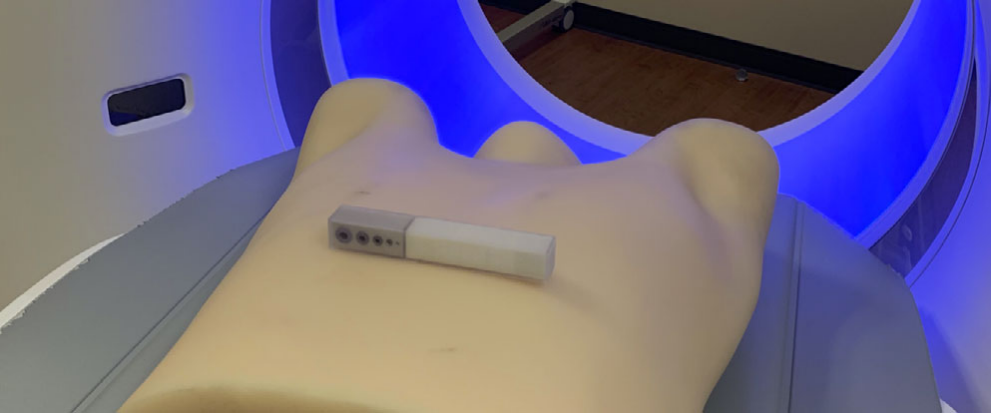
Simulated use of calibration mini‐phantom in clinical CT. Demonstration photograph of the calibration mini‐lung‐phantom placed on top of an anthropomorphic thorax phantom, as an example placement of the mini‐lung‐phantom on the patient chest in clinical chest CT.

We describe the creation and characterization of a calibration CT mini‐lung‐phantom incorporating simulated airways and ground‐glass lung densities. One potential application is for scanning the phantom simultaneously with patients undergoing lung quantitative CT, thus serving as ground truth dimensional and CT number references to assess for and potentially correct for inter‐exam variations. This use complements earlier large lung phantoms such as the airway,^22^ COPDGene, and COPDGene2 phantoms,^10^, ^24^ which are typically used in intermittent scheduled scanner calibrations. The workflow for the inclusion of such a mini‐phantom in clinical thoracic CT scanning is described by the following steps: (a). place the mini‐lung‐phantom on the middle of patient’s chest with orientation as indicated on a device; (b). acquire scout view(s); (c). scan per institutional thoracic CT protocol; (d). remove from the patient, sanitize with standard disinfectant wipe, and replace in a storage container. A photograph with the placement of the mini‐phantom placed on an anthropomorphic thorax phantom, serving as an example application of the mini‐lung‐phantom in clinical chest CT scanning is shown in Fig. 5. Because of its compact size, the addition of the mini‐lung‐phantom is expected to have a negligible impact on the radiation dose and image quality of the patient’s CT scan. Applying the mini‐lung‐phantom to improve the quantification accuracy of lung density and airway measurement is currently under investigation. With known geometric dimensions, measurement error from CT scanning and reconstruction process can be assessed on individual patient^14^ and airway measurement can be potentially improved with correction for partial volume effect.^28^

Micro‐CT scanning demonstrates average variance of airway tube dimensions from design specifications at 0.038 mm is much smaller than the typical resolution limit in clinical CT near 0.6 mm. As expected, dimensional measurements from clinical CT reported a larger average absolute measurement error than from micro‐CT (0.3 mm vs. 0.038 mm, *P* = 0.0049), showing that the largest contribution to clinical dimensional measurement error is from scanner resolution limitations, similar to previously reported scanning of tubes by other clinical CT scanners.^22^ The ability to quantify the degree of measurement error also supports the strategy of using the external calibration mini‐lung‐phantom. The quantified scan‐specific values compared with known true values of the airways tubes and foam block densities may be used to determine calibration formulas, which should make it possible to reduce the error in scan‐specific values, analogous to earlier works with coronary artery calcium quantification.^13^

Three‐dimensional printing is a developing technology with expanding applications in medicine. A variety of techniques such as stereolithography (SLA), material jetting, fused deposition modeling (FDM), and selective laser sintering (SLS) offer different advantages to fit desired applications. Stereolithography was chosen for this work given its high dimensional accuracy, with layer thicknesses possible down to 13 micrometers.^29^ In comparison, FDM techniques, although generally with lower dimensional accuracy, have been employed to create medical constructs with variable density by incorporating radiopaque materials such as iodine, gadolinium, and barium sulfate,^30^, ^31^ although at density ranges that are very different from the relatively much lower normal lung and ground‐glass opacities. Current 3‐D printing’s inability to reliably produce lung ground‐glass densities necessitated the use of polyurethane foams, which are manufactured to specified closer‐to‐air densities and have been validated in prior CT phantoms,^10^, ^23^ To minimize potential effects on image quality and dose, the design of the mini‐lung‐phantom is kept small, at 1.75 × 1.75 × 12 cm, and light at 22 g. The 3‐D printed plastic shell is water and alcohol‐resistant and can be disinfected by a sanitizing wipe between uses.

Three‐dimensional printing of imaging phantoms is being increasingly reported, ranging from simulated low‐contrast soft tissue lesions^32^ to whole thoracic phantoms for radiation treatment planning.^18^ The average dimensional error of the 3‐D printed tubes in this work at 0.038 mm is superior to the average error of comparable tubes made from traditional machining with an average absolute error of 0.11 mm.^22^ As that 3‐D printing in this work was carried out by contract manufacturing by a third party vendor, the capital cost of purchase and maintenance of the 3‐D printer was avoided, and the per‐unit cost of ~$100 for the 3‐D printed portions of each of the mini‐lung‐phantoms is contrasted with $2,000 per machined sample of the prior airway phantom.^22^ Three‐Dimensional printing offers the advantages of rapid prototyping, such that design changes and new features can be relatively quickly incorporated into future iterations, and effective prototypes can be readily translated to production. Thus, the techniques described facilitate the cost‐effective manufacturing and potential dissemination of large numbers of calibration phantoms across institutions.

Numerous factors can introduce uncertainty in CT imaging and quantification.^8^ Recently, it was demonstrated that deep learning image reconstruction algorithms may introduce novel and difficult to detect artifacts on CT and MRI.^33^ With other factors such as evolving scanning protocols and the continued advent of automated detection and quantification software algorithms, the presence of external calibration phantom with established ground truth values included with every scan can help detect inaccuracies and facilitate continuous quality assurance.

Limitations of our study include: first, the relatively simple geometry of the tubes does not fully simulate the complex geometry of the airways tree. Future designs incorporating more complex geometries can be enabled by 3‐D printing. Second, the incorporation of various simulated lung densities necessitated the use of machined polyurethane foam blocks, as that current 3D printed materials are closer to water/plastic/soft tissue densities and cannot accurately re‐create closer‐to‐air lung densities. While polyurethane foam also does not exactly simulate the complex alveolar micro‐anatomy of the lung parenchyma, and edge enhancing reconstruction kernels or iterative reconstruction algorithms could impact foam texture in a very different manner compared to actual parenchymal ground‐glass densities, polyurethane foam is a stable material that has been previously validated for the recreation of lung density in CT phantoms^10^, ^23^ and it is an ideal candidate for the measurement of mean CT number in lung density application. Rodrigues et al. showed the mean CT numbers of lung mimicking foam densities do not fluctuate significantly between conventional FBP and iterative reconstruction methods.^34^ Third, the airway dimension analysis of clinical CT is only performed for one scanner at one dose level, because in this study we intend to focus on the characterization of tubes with a typical clinical CT scanner instead of studying the impact of scanner models and scanning conditions on the airway measurement accuracy. Fourth, although the CT number is considered uniform across the scan field of view, the spatial resolution within a CT image degrades at the off‐center location. Therefore, the periphery location of the mini‐lung‐phantom scanned on the patient’s chest surface may lead to different degrees of blurring between the printed airways and patient’s airways. The impact of this difference and correction approach warrants further research.

## 5 CONCLUSION

In summary, calibration CT mini‐lung‐phantoms incorporating simulated airways and ground‐glass lung densities were created using 3‐D printing and conventional machining. This phantom has the potential to facilitate monitoring and help decrease uncertainty in quantitative lung CT.

## Conflict of Interest

No conflicts of interest.

## Author Contribution

H. Henry Guo.

- Substantial contributions to the conception or design of the work; and the acquisition, analysis, and interpretation of data for the work;
- Drafting the work or revising it critically for important intellectual content;
- Final approval of the version to be published;
- Agreement to be accountable for all aspects of the work in ensuring that questions related to the accuracy or integrity of any part of the work are appropriately investigated and resolved.

Mats Persson.

- Analysis and interpretation of data for the work;
- Drafting the work or revising it critically for important intellectual content;
- Final approval of the version to be published;
- Agreement to be accountable for all aspects of the work in ensuring that questions related to the accuracy or integrity of any part of the work are appropriately investigated and resolved.

Oliver Weinheimer.

- Analysis and interpretation of data for the work;
- Drafting the work or revising it critically for important intellectual content;
- Final approval of the version to be published;
- Agreement to be accountable for all aspects of the work in ensuring that questions related to the accuracy or integrity of any part of the work are appropriately investigated and resolved.

Jarrett Rosenberg.

- Analysis and interpretation of data for the work;
- Drafting the work or revising it critically for important intellectual content;
- Final approval of the version to be published;
- Agreement to be accountable for all aspects of the work in ensuring that questions related to the accuracy or integrity of any part of the work are appropriately investigated and resolved.

Terry E. Robinson.

- Substantial contributions to the conception or design of the work;
- Drafting the work or revising it critically for important intellectual content;
- Final approval of the version to be published;
- Agreement to be accountable for all aspects of the work in ensuring that questions related to the accuracy or integrity of any part of the work are appropriately investigated and resolved.

Jia Wang.

- Substantial contributions to the conception or design of the work; and the acquisition, analysis, and interpretation of data for the work;
- Drafting the work or revising it critically for important intellectual content;
- Final approval of the version to be published;
- Agreement to be accountable for all aspects of the work in ensuring that questions related to the accuracy or integrity of any part of the work are appropriately investigated and resolved.

## 6 DISCLOSURE STATEMENT

“MP is former shareholder in Prismatic Sensors AB and was visiting researcher with General Electric Company in 2019‐2020, funded by the EU Research Executive Agency.” None of these activities are related to the present article.

## References

[1] Chen A , Karwoski RA , Gierada DS , Bartholmai BJ , Koo CW . Quantitative CT analysis of diffuse lung disease. Radiographics. 2020;40:28–43. 31782933 10.1148/rg.2020190099

[2] Galbán CJ , Han MK , Boes JL , et al, Computed tomography–based biomarker provides unique signature for diagnosis of COPD phenotypes and disease progression. Nat Med. 2012;18:1711–1715. 23042237 10.1038/nm.2971 PMC3493851

[3] Bartholmai BJ , Raghunath S , Karwoski RA , et al, Quantitative computed tomography imaging of interstitial lung diseases. J Thorac Imaging. 2013;28:298–307. 23966094 10.1097/RTI.0b013e3182a21969 PMC3850512

[4] Goris ML , Zhu HJ , Blankenberg F , Chan F , Robinson TE . An automated approach to quantitative air trapping measurements in mild cystic fibrosis. Chest. 2003;123:1655–1663. 12740287 10.1378/chest.123.5.1655

[5] Wu X , Kim GH , Salisbury ML , et al, Computed tomographic biomarkers in idiopathic pulmonary fibrosis. The future of quantitative analysis. Am J Respir Crit Care Med. 2019;199:12–21. 29986154 10.1164/rccm.201803-0444PP

[6] Han MK , Kazerooni EA , Lynch DA , et al, Chronic obstructive pulmonary disease exacerbations in the COPDGene study: associated radiologic phenotypes. Radiology. 2011;261:274–282. 21788524 10.1148/radiol.11110173 PMC3184233

[7] Colombi D , Bodini FC , Petrini M , et al, Well‐aerated lung on admitting chest CT to predict adverse outcome in COVID‐19 pneumonia. Radiology. 2020;296:E86–E96. 32301647 10.1148/radiol.2020201433 PMC7233411

[8] Fletcher JG , Leng S , Yu L , McCollough CH . Dealing with uncertainty in CT images. Radiology. 2016;279:5–10. 26989927 10.1148/radiol.2016152771

[9] McCollough CH , Chen GH , Kalender W , et al, Achieving routine submillisievert CT scanning: report from the summit on management of radiation dose in CT. Radiology. 2012;264:567–580. 22692035 10.1148/radiol.12112265 PMC3401354

[10] Sieren JP , Newell JD , Judy PF , et al, Reference standard and statistical model for intersite and temporal comparisons of CT attenuation in a multicenter quantitative lung study. Med Phys. 2012;39:5757–5767. 22957640 10.1118/1.4747342 PMC3448623

[11] Mets OM , de Jong PA , van Ginneken B , Gietema HA , Lammers JW . Quantitative computed tomography in COPD: possibilities and limitations. Lung. 2012;190:133–145. 22179694 10.1007/s00408-011-9353-9 PMC3310986

[12] Hammond E , Sloan C , Newell JD , et al, Comparison of low‐ and ultralow‐dose computed tomography protocols for quantitative lung and airway assessment. Med Phys. 2017;44:4747–4757. 28657201 10.1002/mp.12436 PMC5603212

[13] Nelson JC , Kronmal RA , Carr JJ , et al, Measuring coronary calcium on CT images adjusted for attenuation differences. Radiology. 2005;235:403–414. 15858082 10.1148/radiol.2352040515

[14] Henschke CI , Yankelevitz DF , Yip R , et al, Tumor volume measurement error using computed tomography imaging in a phase II clinical trial in lung cancer. J Med Imaging (Bellingham, Wash). 2016;3:035505. 10.1117/1.JMI.3.3.035505 PMC5028411 27660808

[15] Mitsouras D , Liacouras P , Imanzadeh A , et al, Medical 3D printing for the radiologist. Radiographics. 2015;35:1965–1988. 26562233 10.1148/rg.2015140320 PMC4671424

[16] Matsumoto JS , Morris JM , Foley TA , et al, Three‐dimensional physical modeling: applications and experience at Mayo Clinic. Radiographics. 2015;35:1989–2006. 26562234 10.1148/rg.2015140260

[17] Giannopoulos AA , Steigner ML , George E , et al, Cardiothoracic applications of 3‐dimensional printing. J Thorac Imaging. 2016;31:253–272. 27149367 10.1097/RTI.0000000000000217 PMC4993676

[18] Hazelaar C , van Eijnatten M , Dahele M , et al, Using 3D printing techniques to create an anthropomorphic thorax phantom for medical imaging purposes. Med Phys. 2018;45:92–100. 29091278 10.1002/mp.12644

[19] Jahnke P , Limberg FRP , Gerbl A , et al, Radiopaque three‐dimensional printing: a method to create realistic CT phantoms. Radiology. 2017;282:569–575. 27626676 10.1148/radiol.2016152710

[20] Weibel ER , Gomez DM . Architecture of the human lung: use of quantitative methods establishes fundamental relations between size and number of lung structures. Science. 1962;137:577–585. 14005590 10.1126/science.137.3530.577

[21] Reinhardt JM , D'Souza ND , Hoffman EA . Accurate measurement of intrathoracic airways. IEEE Trans Med Imaging. 1997;16:820–827. 9533582 10.1109/42.650878

[22] Robinson TE , Long FR , Raman P , et al, An airway phantom to standardize CT acquisition in multicenter clinical trials. Academic radiology. 2009;16:1134–1141. 19467609 10.1016/j.acra.2009.02.018

[23] Levine ZH , Li M , Reeves AP , et al, A low‐cost density reference phantom for computed tomography. Med Phys. 2009;36:286–288. 19291968 10.1118/1.3049596 PMC2673671

[24] Chen‐Mayer HH , Fuld MK , Hoppel B , et al, Standardizing CT lung density measure across scanner manufacturers. Med Phys. 2017;44:974–985. 28060414 10.1002/mp.12087 PMC6276120

[25] Achenbach T , Weinheimer O , Biedermann A , et al, MDCT assessment of airway wall thickness in COPD patients using a new method: correlations with pulmonary function tests. Eur Radiol. 2008;18:2731–2738. 18641993 10.1007/s00330-008-1089-4

[26] Weinheimer O , Achenbach T , Bletz C , Duber C , Kauczor HU , Heussel CP . About objective 3‐d analysis of airway geometry in computerized tomography. IEEE Trans Med Imaging. 2008;27:64–74. 18270063 10.1109/TMI.2007.902798

[27] Jobst BJ , Weinheimer O , Buschulte T , et al, Longitudinal airway remodeling in active and past smokers in a lung cancer screening population. Eur Radiol. 2019;29:2968–2980. 30552475 10.1007/s00330-018-5890-4

[28] Conradi SH , Lutey BA , Atkinson JJ , Wang W , Senior RM , Gierada DS . Measuring small airways in transverse CT images correction for partial volume averaging and airway tilt. Academic radiology. 2010;17:1525–1534. 20947386 10.1016/j.acra.2010.08.005 PMC2975772

[29] 3D Systems Product technical specification . 3D Systems, Inc.; 2018.

[30] Ballard DH , Jammalamadaka U , Tappa K , et al, 3D printing of surgical hernia meshes impregnated with contrast agents: in vitro proof of concept with imaging characteristics on computed tomography. 3D Print Med. 2018;4:13. 30649673 10.1186/s41205-018-0037-4 PMC6283811

[31] Hamedani BA , Melvin A , Vaheesan K , Gadani S , Pereira K , Hall AF . Three‐dimensional printing CT‐derived objects with controllable radiopacity. J Appl Clin Med Phys. 2018;19:317–328. 29411529 10.1002/acm2.12278 PMC5849815

[32] Solomon J , Ba A , Bochud F , Samei E . Comparison of low‐contrast detectability between two CT reconstruction algorithms using voxel‐based 3D printed textured phantoms. Med Phys. 2016;43:6497. 27908164 10.1118/1.4967478

[33] Antun V , Renna F , Poon C , Adcock B , Hansen AC . On instabilities of deep learning in image reconstruction and the potential costs of AI. Proc Natl Acad Sci. 2020;117:30088–30095. 32393633 10.1073/pnas.1907377117 PMC7720232

[34] Rodriguez A , Ranallo FN , Judy PF , Fain SB . The effects of iterative reconstruction and kernel selection on quantitative computed tomography measures of lung density. Med Phys. 2017;44:2267–2280. 28376262 10.1002/mp.12255 PMC5497316

